# Cognitive improvement by non-pharmacological electrical stimulation modalities in mild cognitive impairment: a protocol for systematic review and network meta-analysis

**DOI:** 10.3389/fnagi.2026.1752516

**Published:** 2026-03-11

**Authors:** Tao Zhu, Luyao Cai, Lang Hu, Dan Yang, Ming Li, Fei Quan, Chunxia Lu, Shengdong Liu, Jin Cui

**Affiliations:** 1 School of Acupuncture-Moxibustion and Tuina, Guizhou University of Traditional Chinese Medicine, Guiyang, China; 2 Department of Rehabilitation, The First Affiliated Hospital of Guizhou University of Traditional Chinese Medicine, Guiyang, China; 3 Department of Acupuncture and Moxibustion, The First Affiliated Hospital of Guizhou University of Traditional Chinese Medicine, Guiyang, China

**Keywords:** electrical stimulation, mild cognitive impairment, network meta-analysis, protocol, systematic review

## Abstract

**Objective:**

Mild cognitive impairment, characterized by progressive cognitive decline, represents a prevalent transitional state among the global aging population and demonstrates high conversion rates to Alzheimer’s disease, establishing itself as a critical window for preventive interventions against AD. Although growing evidence supports the efficacy of various non-pharmacological therapies in enhancing cognitive function, their comparative effectiveness remains insufficiently elucidated. This study aims to analyze the efficacy and safety of different electrical stimulation modalities in treating MCI patients, quantitatively compare the therapeutic benefits across multiple interventions, and provide evidence-based recommendations to facilitate informed clinical decision-making.

**Methods:**

We will systematically search 13 databases. All relevant studies published from inception until November 1, 2025, will be retrieved. Two reviewers will independently assess the risk of bias for all included studies using the revised Cochrane Risk of Bias tool (RoB 2). The primary outcome will be the Montreal Cognitive Assessment score to evaluate changes in cognitive function. Secondary outcomes will include neuropsychological assessments related to cognition, such as the Mini-Mental State Examination (MMSE) and the Alzheimer’s Disease Assessment Scale–Cognitive Subscale (ADAS-Cog), as well as the modified Barthel Index for activities of daily living and the patient-reported Pittsburgh Sleep Quality Index. Data synthesis will be performed using Stata software, employing a random-effects network meta-analysis model to compare the efficacy and safety of non-pharmacological electrical stimulation therapies. The surface under the cumulative ranking curve (SUCRA) will be used to estimate the probability of intervention hierarchies. The strength of evidence will be evaluated using the Grading of Recommendations, Assessment, Development, and Evaluations framework.

**Conclusion:**

This study will synthesize evidence from multiple studies on various electrical stimulation therapies for improving cognitive function in patients with mild cognitive impairment, thereby providing a diverse body of evidence to support clinical decision-making by physicians and optimization of treatment strategies for patients.

**Study Protocols Registration:**

[https://www.crd.york.ac.uk/prospero/], identifier [CRD420251184505].

## 1 Introduction

Mild cognitive impairment (MCI) refers to an early clinical syndrome and an intermediate stage between normal cognitive aging and dementia. It is characterized by objectively measured cognitive impairment that is greater than expected for the patient’s age, without evident significant impairment in complex instrumental activities of daily living (Sanford, 2017). In recent years, the incidence of MCI increases with age, with prevalence rates ranging from 6.7% to 14.8% among individuals aged 60–79 years and reaching 25.2% in those aged 80–84 years. Furthermore, a 2-years follow-up study of MCI patients aged 65 and older revealed a cumulative dementia conversion rate of 14.9% (Petersen et al., 2018). Another epidemiological study has reported that among the global population aged 60 years and older, the prevalence of MCI ranges from 3% to 42%, with an annual incidence rate of 21.5 to 71.3 per 1,000 persons (Ward et al., 2012). MCI is considered a prodromal phase of Alzheimer’s disease (AD), carrying a significantly elevated risk for progression to full dementia. Research has established that 10%–15% of MCI patients convert to AD annually, a rate far exceeding that observed in the healthy population (Lissek and Suchan, 2021). The World Alzheimer Report 2021 highlighted that MCI, which can be stable or even reversible, represents a critical window for the secondary prevention of dementia (Dubois et al., 2021). Thus, MCI constitutes a critical period for early preventive intervention. This makes it imperative to identify effective and safe measures that can improve cognitive function, slow its decline, and ultimately enhance the quality of life among the elderly.

Currently, the management of Mild Cognitive Impairment primarily includes pharmacological and non-pharmacological approaches. Pharmacological therapies lack sufficient evidence for recommended use (Cohen et al., 2023; Larson, 2018). Among non-pharmacological interventions, electrical stimulation is one of the most commonly used methods for MCI patients, with diverse types and broad application, and has been shown to significantly improve overall cognitive function (Gomes et al., 2019; Jiang et al., 2020; Wang L. F. et al., 2022). Currently, electrical stimulation techniques employed in the cognitive domain include transcranial direct current stimulation (tDCS) (Hausman et al., 2023), transcranial alternating current stimulation (tACS) (Tang et al., 2024), transcutaneous electrical acupoint stimulation (TEAS) (Wang L. F. et al., 2022), repetitive transcranial magnetic stimulation (rTMS) (Gao et al., 2023), electroacupuncture (Choi et al., 2021), cortical electrical stimulation (Kuo et al., 2021), deep brain stimulation (DBS) (Ni et al., 2025), neuromuscular electrical stimulation (NMES) (Vints et al., 2024), and transcutaneous auricular vagus nerve stimulation (taVNS) (Wang L. F. et al., 2022). Preliminary evidence suggests that electrical stimulation can exert various effects on the brain by targeting specific brain regions and associated networks, and modulating neural circuits–including the default mode network (DMN) and cognitive control network (CCN) (He et al., 2021; Tseng et al., 2023). It also continuously regulates neuronal excitability and connectivity, thereby improving cognitive function (Huang et al., 2017). Furthermore, the modulation of cortical excitability constitutes a key mechanism through which electrical stimulation improves cognitive behavior. By applying weak currents via scalp electrodes to targeted brain regions, this technique modulates endogenous cortical oscillations, rectifies oscillatory abnormalities in MCI patients, and restores cognitive and memory-related oscillatory patterns (Benussi et al., 2021; Kim et al., 2021; Nissim et al., 2023). Neural or cortical oscillations refer to rhythmic fluctuations in the local field potentials generated by inputs from tens of thousands of neurons (Zoefel et al., 2018). This oscillatory activity underlies information transfer in patterns of neural activity within brain networks associated with behavior and memory (Hanslmayr et al., 2019). It encompasses multiple frequency bands, each playing a crucial role in cognitive and memory processes. In particular, gamma-frequency oscillations have been demonstrated to be critical for higher-order cognitive functions (Reinhart and Nguyen, 2019; Traikapi and Konstantinou, 2021).

In addition to modulating neural networks and cortical excitability, alterations in biological systems are recognized as a key strategy for managing MCI. Prominent among these are pro-inflammatory markers such as interleukin-6 (IL-6) and CX3CL1, as well as brain-derived neurotrophic factor (BDNF), which have been implicated in the pathophysiology of MCI (Salech et al., 2022). The presence of activated microglia and astrocytes in patients with cognitive impairment signifies an activated inflammatory response and indicates neuronal damage or even death (van Olst et al., 2025). These inflammatory markers provide insight into neurodegenerative processes; their elevated levels indicate the presence of neuroinflammation, which is often associated with cognitive decline (Pawelec et al., 2020; Salech et al., 2022). Furthermore, the cholinergic system is critically involved in cognitive processes. Reduced cholinergic activity, along with impairments in acetylcholine (ACh) and various cholinergic receptors, can lead to deficits in memory and cognitive function (Wu et al., 2010). Finally, oxidative stress and free radical damage are closely associated with cognitive impairment (Chico et al., 2013). In the MCI stage, brain tissue exhibits a marked state of oxidative stress, characterized by significantly elevated levels of protein carbonyls, 3-nitrotyrosine, and lipid peroxidation markers–including 4-hydroxy-2-non-enal, F2-isoprostanes, F4-neuroprostanes, and malondialdehyde (Butterfield, 2011; Feng and Wang, 2012). Emerging evidence has demonstrated that electrical stimulation can suppress neuroinflammatory responses, exert anti-inflammatory effects (Alcala-Lozano et al., 2025), modulate cholinergic neurons (Zhou et al., 2022), mitigate oxidative stress damage by upregulating antioxidant expression (Wang et al., 2024), and thereby improve cognitive function in patients.

To date, only one network meta-analysis has indicated that different electrical stimulation interventions confer distinct cognitive benefits in patients with MCI, with repetitive transcranial magnetic stimulation (rTMS) demonstrating the most beneficial effects on cognition and language (Liu et al., 2024). Another meta-analysis demonstrated that non-invasive brain stimulation (NIBS) is an effective and safe therapeutic approach for patients with MCI or AD, and its cognitive benefits may be mediated by the positive modulation of spontaneous neural activity and cognitive networks (Wang et al., 2023). It is noteworthy that the electrical stimulation modalities included in these two studies were incomplete. Specifically, widely used clinical interventions such as electroacupuncture and transcutaneous electrical acupoint stimulation were not considered. Furthermore, both studies are limited by small sample sizes in the included randomized controlled trials, substantial heterogeneity in outcomes, and relatively short follow-up durations. Moreover, a review of the current literature reveals that no study has yet systematically evaluated the efficacy and safety of multiple electrical stimulation techniques–including non-invasive brain stimulation, electroacupuncture, and transcutaneous electrical acupoint stimulation–for treating MCI, nor quantitatively analyzed their relative advantages or potential benefits for patients.

In medical research, conventional meta-analysis is limited to direct comparisons between two interventions, thereby restricting its analytical scope. In contrast, network meta-analysis represents a novel methodology that integrates both direct and indirect evidence. This approach enables researchers to synthesize data from multiple randomized controlled trials, estimate the probability of each intervention’s relative efficacy, and ultimately rank and compare different treatment strategies (Rouse et al., 2017; Yang et al., 2022). Leveraging this methodological advancement, we will conduct a systematic review and network meta-analysis to evaluate the efficacy and safety of various electrical stimulation therapies for MCI. By quantitatively assessing their relative advantages and potential benefits, this study aims to provide clinicians and patients with an evidence-based clinical foundation to guide treatment decision-making.

## 2 Methods

### 2.1 Objectives

(1) ()Efficacy Evaluation: This study will employ systematic review and network meta-analysis methodologies to quantitatively assess the therapeutic efficacy of various non-pharmacological electrical stimulation therapies for mild cognitive impairment. It aims to elucidate the advantages and limitations of different intervention strategies in improving cognitive function, thereby providing robust scientific evidence to support clinical decision-making.
(2) ()Safety Evaluation: This study will systematically collate and analyze the incidence of adverse events associated with non-pharmacological electrical stimulation modalities for MCI and conduct safety assessments. It will evaluate the safety of these stimulation techniques in clinical practice to provide clinicians and patients with reliable safety information.

### 2.2 Research registration

This systematic review protocol was rigorously developed in full accordance with the Preferred Reporting Items for Systematic Reviews and Meta-Analyses Protocols (PRISMA-P) guidelines (Shamseer et al., 2015) and the PRISMA extension for network meta-analyses (Hutton et al., 2015). To ensure methodological transparency and reproducibility, the protocol has been registered prospectively in the International Prospective Register of Systematic Reviews (PROSPERO; registration number: CRD420251184505). The completed PRISMA-P checklist is provided as Supplementary Appendix 1.

### 2.3 Search strategy

A comprehensive search strategy will be employed, combining Medical Subject Headings (MeSH) and free-text terms to capture studies related to mild cognitive impairment (MCI), electrical stimulation, and cognitive function. Boolean operators (OR, AND) will be used to refine the search. The search protocol is designed to include all available literature from the inception of each database until 1 November 2025. The following electronic databases will be systematically searched: PubMed, Embase, the Cochrane Library, Web of Science, Scopus, ClinicalTrials.gov, the WHO International Clinical Trials Registry Platform (WHO-ICTRP), OpenGrey, ProQuest, China National Knowledge Infrastructure (CNKI), VIP Database, WanFang Database, and the Chinese Biomedical Literature Database (CBM). Additionally, the reference lists of all included studies, along with relevant clinical trial reports and review articles, will be manually screened. A manual search will also be conducted through specialized journal collections, bibliographies, and conference proceedings focusing on mild cognitive impairment and electrical stimulation. Using the PubMed database as an example, the following English search terms were employed: (Mild cognitive impairment OR Cognitive impairment OR Cognitive decline OR cognitive impairment OR Cognitive dysfunction OR MCI) AND (Electrical stimulation OR Electrical Stimulation OR Electrical Stimulations OR Stimulation, Electrical OR Stimulations, Electrical OR Stimulation, Electric OR Electric Stimulations OR Stimulations, Electric OR transcranial direct current stimulation OR transcranial alternating current stimulation OR transcutaneous electrical acupoint stimulation OR repetitive transcranial magnetic stimulation OR electroacupuncture OR cortical electrical stimulation OR deep brain stimulation OR neuromuscular electrical stimulation OR transcutaneous auricular vagus nerve stimulation) AND (RCT OR randomized controlled trial OR randomized OR placebo). The search strategy for each database was tailored to its specific features. The electronic search strategy was developed and verified by two reviewers in accordance with the PRESS checklist to ensure its comprehensiveness, accuracy and standardization. The literature search was conducted independently by two researchers, and any discrepancies were resolved through discussion or adjudicated by a third reviewer to minimize search bias. The detailed search strategies for both Chinese and English databases are provided in Supplementary Appendix 2.

### 2.4 Study selection criteria

#### 2.4.1 Types of participants

Participants will include individuals diagnosed with mild cognitive impairment according to any recognized diagnostic criteria, encompassing both amnestic and non-amnestic subtypes, with diagnostic codes corresponding to mild cognitive impairment identifiers in the International Classification of Diseases. The study will impose no restrictions regarding participants’ sex, age, occupation, or educational background. Individuals exhibiting cognitive impairment attributable to other causes–such as neurological disorders, tumors, or post-surgical sequelae–will be excluded.

#### 2.4.2 Types of interventions

The intervention group must administer electrical stimulation therapy to participants, including, but not limited to, transcranial direct current stimulation (tDCS), transcranial alternating current stimulation (tACS), transcutaneous electrical acupoint stimulation (TEAS), repetitive transcranial magnetic stimulation (rTMS), electroacupuncture, cortical electrical stimulation, deep brain stimulation (DBS), neuromuscular electrical stimulation (NMES), and transcutaneous auricular vagus nerve stimulation (taVNS). Additionally, the combination of multiple forms of electrical stimulation is permitted, with no restrictions imposed on treatment duration or follow-up periods.

#### 2.4.3 Types of control groups

For the control group, a three-tiered hierarchical framework was employed according to the network connectivity requirements of the network meta-analysis, aiming to reduce heterogeneity and ensure analytical validity. The first tier permits the use of pharmacologically inert placebos and sham stimulation procedures; the second tier allows guideline-recommended conventional treatment regimens; and the third tier includes active interventions known to exceed conventional care, provided they explicitly exclude any form of electrical stimulation and are included only when direct comparative evidence is available to maintain network connectivity. All control interventions across these three tiers strictly prohibit the administration of any form of electrical stimulation therapy.

#### 2.4.4 Types of outcomes

##### 2.4.4.1 Primary outcomes

The primary outcome of this study was the assessment of global cognitive function improvement following electrical stimulation therapy for MCI, evaluated using the Montreal Cognitive Assessment (MoCA) (Islam et al., 2023). Previous studies have demonstrated that the MoCA is commonly used to assess cognitive function in individuals with MCI, evaluating multiple cognitive domains including visuospatial and executive functions, naming, delayed recall, attention, language abilities, abstract thinking, calculation, and orientation. The MoCA has a total possible score of 30, wherein lower scores indicate more severe cognitive impairment.

##### 2.4.4.2 Secondary outcomes

Other neuropsychological assessment scores related to cognition, such as the Mini-Mental State Examination (MMSE) and the Alzheimer’s Disease Assessment Scale-Cognitive Subscale (ADAS-Cog), will serve as secondary outcomes. Additionally, secondary outcomes will include assessments of activities of daily living and patient-reported outcomes to provide a more comprehensive evaluation of intervention effects, such as the modified Barthel Index, Pittsburgh Sleep Quality Index, and safety assessments.

#### 2.4.5 Types of studies

This review will include only published, peer-reviewed randomized controlled trials (RCTs), with no restrictions imposed on language or regional origin. Studies employing non-randomized designs, case-control studies, cohort studies, case reports, review articles, preprints, conference abstracts, and study protocols will be excluded from the analysis. Additionally, duplicate publications will be excluded, retaining only the study with the largest sample size in cases of population or dataset overlap.

### 2.5 Study selection

**FIGURE 1:**
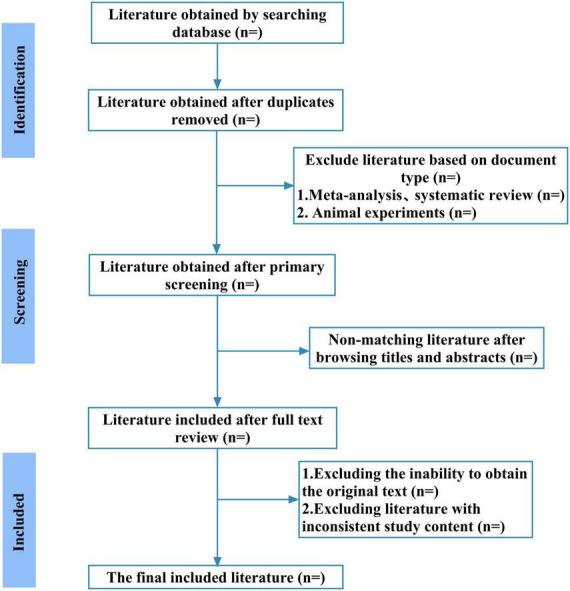
The details of the selection process.

In this study, two independent reviewers will independently perform literature searches and screening according to the search strategy. EndNote 20 will be used for reference management. After removing duplicates, the reviewers will sequentially screen titles, abstracts, and keywords, followed by a full-text assessment to identify literature meeting the inclusion criteria. Any disagreements will be resolved by a third reviewer. Reasons for exclusion will be systematically recorded, and the screening workflow is presented in the PRISMA flow diagram in Figure 1.

### 2.6 Data extraction

In this study, two independent data managers extracted relevant data from all finally included studies using standardized Excel forms. When insufficient or missing data were encountered during extraction, we contacted the corresponding authors via email to obtain complete information. All extracted data underwent rigorous cross-checking prior to analysis, and any discrepancies were discussed within the research team to ensure data accuracy. The extracted data included the following:

Study characteristics: author, publication year, country, study design, study centers.

Participant characteristics: sample size, sex, age, diagnostic criteria, educational level, disease features (including disease duration and amnesic/non-amnesic subtypes), medication use, treatment duration, and follow-up duration.

Intervention and control measures: type of electrical stimulation, stimulation site(s), treatment duration, frequency, treatment course, and key stimulation parameters;

Outcome measures: all reported outcomes from the included studies, quality of life assessments, records of adverse events, and time points of each measurement.

### 2.7 Risk of bias assessment

Two reviewers will assess the risk of bias for all included studies using the Cochrane Risk of Bias tool (RoB 2) (Sterne et al., 2019). The RoB 2 tool evaluates five domains: bias arising from the randomization process, bias due to deviations from intended interventions, bias due to missing outcome data, bias in measurement of the outcome, and bias in selection of the reported result. Each domain is categorized as “low risk of bias,” “some concerns,” or “high risk of bias.” To minimize subjective discrepancies between reviewers, all reviewers received rigorous training based on detailed guidelines prior to the formal assessment. Any disagreements in the assessments were resolved through discussion with a third reviewer within the evaluation panel.

### 2.8 Data synthesis

#### 2.8.1 Pairwise meta-analysis

Statistical analyses in this study were conducted using Stata software (version 14.0; Stata Corporation, College Station, Texas, USA). The MoCA score was treated as a continuous variable, with the standardized mean difference and 95% confidence interval employed as the summary statistic. Heterogeneity was quantified using the *I*^2^ statistic alongside the *p*-value, with a *p*-value < 0.05 considered statistically significant. When *I*^2^ was ≤50%, a fixed-effects model was applied to estimate the pooled effect size; when *I*^2^ exceeded 50%, a random-effects model was used, indicating substantial heterogeneity. In such cases, the potential practical sources of heterogeneity were further explored, and their possible impact on the study findings was discussed.

#### 2.8.2 Network meta-analysis

For the network meta-analysis, a random-effects model will be adopted under the frequentist framework to synthesize both direct and indirect evidence across all interventions. Indirect comparisons will be based on the transitivity assumption, whereby the unknown difference between A and B is inferred from known differences of A versus C and B versus C. To verify the robustness of the transitivity assumption and explore sources of heterogeneity, subgroup analyses or meta-regression based on effect modifiers (e.g., baseline patient characteristics, intervention parameters) will be performed. For dichotomous outcomes, risk ratios (RR) will be used; for continuous outcomes, mean differences (MD) or standardized mean differences (SMD) will be calculated, with all effect estimates reported together with their 95% confidence intervals (CI).

First, we will use the “network plot” command to generate a network diagram, illustrating the quantitative relationships between studies and interventions. In this diagram, each node represents a distinct intervention, and the edges between nodes denote “direct comparison evidence” between pairs of interventions. The size of each node is proportional to the corresponding sample size, with larger nodes indicating a greater sample size supporting that intervention. Additionally, we will employ the “mvmeta inconsistency” global consistency test to evaluate the agreement between direct and indirect comparison evidence. If *P* > 0.05, it suggests no significant conflict between direct and indirect evidence, allowing all evidence to be pooled for subsequent efficacy comparisons; if *P* < 0.05, it indicates substantial inconsistency, necessitating further investigation into potential causes. When closed loops are present in the network diagram, the “network sidesplit all, tau” command will be applied to conduct node-splitting tests for local inconsistency identification. Subsequently, a random-effects model for network meta-analysis was employed to synthesize the data and compare the relative efficacy of different electrical stimulation therapies. Finally, interventions were ranked using the “sucra prob*” command, accompanied by cumulative probability plots. The surface under the cumulative ranking curve (SUCRA) was calculated to estimate the probability of each intervention being optimal, with higher SUCRA scores indicating a greater likelihood of superior efficacy. Additionally, publication bias and small-study effects across the included studies were assessed using funnel plots generated by the “netfunnel” command. All study results were presented visually.

#### 2.8.3 Subgroup analysis

This study aims to systematically explore and mitigate potential sources of heterogeneity in the network meta-analysis through pre-specified subgroup analyses based on effect modifiers. Stratification will be performed based on prespecified effect modifiers, including patient characteristics (e.g., different diagnostic criteria, age, amnestic/non-amnestic subtype, years of education) and intervention types (e.g., different modalities of electrical stimulation therapy). Subgroup analysis of participant stratification will assess the influence of population-specific characteristics on intervention outcomes, while analysis of intervention types will elucidate the differential efficacy and safety profiles of various electrical stimulation therapies.

### 2.9 Sensitivity analysis

We will perform sensitivity analysis using the leave-one-out method, wherein each study will be sequentially excluded and the model will be reconstructed to calculate the change in the SUCRA (surface under the cumulative ranking curve) values for each intervention. A change of less than 15% will be considered indicative of robust results (Daly et al., 2019). If substantial heterogeneity is detected in the network (*I*^2^ > 50%), we will first explore its potential sources through subgroup analyses based on the type of electrical stimulation (e.g., transcranial direct current stimulation, transcranial alternating current stimulation, repetitive transcranial magnetic stimulation). For subgroups containing a sufficient number of studies, we will consider conducting meta-regression to examine the relationship between specific stimulation parameters (e.g., frequency, intensity) and effect sizes, provided these parameters are defined consistently within that modality. If the number of studies is insufficient for meta-regression, these parameters will be summarized descriptively and their potential influence will be addressed in the section “3 Discussion” as a limitation.

### 2.10 Publication bias

This study will systematically evaluate publication bias using funnel plots, Egger’s regression test, and Begg’s test when at least 10 studies are included. The funnel plot method provides a graphical assessment of potential publication bias by plotting effect sizes against their corresponding standard errors, where asymmetry in the distribution of studies suggests possible bias. Complementing this visual approach, Egger’s linear regression test quantitatively assesses publication bias through weighted regression analysis to determine whether the regression intercept significantly deviates from zero. It should be noted that both Begg’s and Egger’s tests may exhibit limited statistical power when applied to small study samples (*n* < 10). Nevertheless, the combined application of funnel plot analysis with Egger’s and Begg’s tests provides a comprehensive and reliable methodology for evaluating publication bias in meta-analyses (Chaimani et al., 2013; Egger et al., 1997).

### 2.11 Grading the quality of evidence

We assessed the certainty of evidence for each outcome using the Grading of Recommendations, Assessment, Development, and Evaluations (GRADE) framework (Balshem et al., 2011). Evidence ratings were downgraded based on five predefined domains: risk of bias, inconsistency, indirectness, imprecision, and publication bias. The strength of evidence was categorized into four levels: high, moderate, low, and very low. High-quality evidence indicates that the true effect is likely close to the estimated effect and can be strongly recommended in decision-making for clinical practice. Conversely, very low-quality evidence suggests that the true effect is likely substantially different from the estimated effect (Puhan et al., 2014).

### 2.12 Ethics and dissemination

Our study exclusively utilizes data obtained from publicly accessible databases and does not directly involve human subjects or public participation in any phase of the research. As all data are derived from previously published literature, this study does not require formal ethics approval from an institutional review board. The final research findings will undergo rigorous peer review and be published in reputable scientific journals.

## 3 Discussion

Mild cognitive impairment (MCI) represents an intermediate state of cognitive decline between normal aging and dementia, categorized as an early clinical syndrome. Consequently, the MCI stage has been widely recognized as the prodromal phase of Alzheimer’s disease, providing a critical window for implementing effective preventive strategies to delay disease onset. Non-pharmacological therapies are recommended as a cornerstone of clinical management for MCI, among which electrical stimulation represents a widely adopted intervention with established efficacy. Previous meta-analyses have demonstrated that electrical stimulation yields superior therapeutic outcomes compared to non-electrical stimulation approaches (Liu et al., 2024; Wang et al., 2023). In this study, multiple non-pharmacological electrical stimulation interventions were included, involving Traditional Chinese Medicine (TCM)-related electrical stimulation modalities such as electroacupuncture and transcutaneous electrical acupoint stimulation. These interventions may introduce clinical heterogeneity due to substantial differences from conventional non-invasive brain stimulation techniques (tDCS, tACS, rTMS) in terms of their theoretical basis, acupoint selection, stimulation frequency, and treatment duration. Furthermore, considerable variability in stimulation parameters also exists within the field of non-invasive brain stimulation techniques (tDCS, tACS, rTMS) themselves. Addressing these differences in the statistical analysis has become a key focus, a challenge, and a methodological difficulty for this network meta-analysis. Therefore, to explore and control for potential heterogeneity, this study has established a rigorous procedure incorporating subgroup analysis, sensitivity analysis, and publication bias analysis. Prespecified subgroup analyses will be conducted based on intervention type, stratifying according to stimulation modality (non-invasive brain stimulation: tDCS, tACS, rTMS; TCM-related electrical stimulation: electroacupuncture, transcutaneous electrical acupoint stimulation). Additionally, if significant heterogeneity (*I*^2^ > 50%) is detected in the analysis results and a sufficient number of studies are available, meta-regression will be employed to explore the sources of heterogeneity. If the number of studies is insufficient, a descriptive summary of the employed stimulation parameters will be provided, the results will be interpreted cautiously, and their potential impact on the findings will be discussed in the section “3 Discussion,” while acknowledging this as a limitation of the current evidence base.

In another aspect, the invasive stimulation techniques included in this study (such as deep brain stimulation and cortical stimulation) differ substantially from non-invasive electrical stimulation in terms of invasiveness, administration procedure, mechanisms of action, target populations, and clinical contexts. To ensure consistency with the transitivity assumption, invasive and non-invasive interventions will not be combined within the same network analysis. Instead, separate network meta-analyses will be constructed for non-invasive stimulation and invasive stimulation, respectively. If the number of studies on invasive stimulation is insufficient, only a descriptive summary will be provided. Finally, although this study analyzes published randomized controlled trials from multiple databases, the exclusion of unpublished studies may introduce potential publication bias, as papers with statistically significant or positive results are more likely to be published, while null or negative findings may be more difficult to publish. This should be regarded as an important limitation when interpreting the study findings. This study will comprehensively synthesize evidence through a systematic review and network meta-analysis (NMA) to evaluate the relative efficacy and safety profiles of all available electrical stimulation therapies for MCI. By integrating existing evidence, it aims to provide evidence-based support for the effectiveness of electrical stimulation in improving cognitive function in MCI, thereby informing clinical decision-making and cognitive intervention strategies.

## References

[B1] Alcala-Lozano R. Carmona-Hernandez R. Ocampo-Romero A. G. Sosa-Millan A. L. Morelos-Santana E. D. Abarca D. Z. (2025). Predicting the beneficial effects of cognitive stimulation and transcranial direct current stimulation in amnestic mild cognitive impairment with clinical, inflammation, and human microglia exposed to serum as potential markers: A double-blind placebo-controlled randomized clinical trial. *Int. J. Mol. Sci.* 26:1754. 10.3390/ijms26041754 40004217 PMC11855719

[B2] Balshem H. Helfand M. Schunemann H. J. Oxman A. D. Kunz R. Brozek J. (2011). Grade guidelines: 3. Rating the quality of evidence. *J. Clin. Epidemiol.* 64 401–406. 10.1016/j.jclinepi.2010.07.015 21208779

[B3] Benussi A. Cantoni V. Cotelli M. S. Cotelli M. Brattini C. Datta A. (2021). Exposure to gamma tacs in alzheimer’s disease: A randomized, double-blind, sham-controlled, crossover, pilot study. *Brain Stimul.* 14 531–540. 33762220 10.1016/j.brs.2021.03.007

[B4] Butterfield D. A. (2011). Oxidative stress in alzheimer disease: Synergy between the butterfield and markesbery laboratories. *Neuromol. Med.* 13 19–22. 10.1007/s12017-010-8123-9 20596797 PMC3035764

[B5] Chaimani A. Higgins J. P. Mavridis D. Spyridonos P. Salanti G. (2013). Graphical tools for network meta-analysis in stata. *PLoS One* 8:e76654. 10.1371/journal.pone.0076654 24098547 PMC3789683

[B6] Chico L. Simoncini C. Lo G. A. Rocchi A. Petrozzi L. Carlesi C. (2013). Oxidative stress and apo e polymorphisms in alzheimer’s disease and in mild cognitive impairment. *Free Radic. Res.* 47 569–576. 10.3109/10715762.2013.804622 23668794

[B7] Choi Y. Jung I. C. Kim A. R. Park H. J. Kwon O. Lee J. H. (2021). Feasibility and effect of electroacupuncture on cognitive function domains in patients with mild cognitive impairment: A pilot exploratory randomized controlled trial. *Brain Sci.* 11:756. 10.3390/brainsci11060756 34200354 PMC8228462

[B8] Cohen S. van Dyck C. H. Gee M. Doherty T. Kanekiyo M. Dhadda S. (2023). Lecanemab clarity ad: Quality-of-life results from a randomized, double-blind phase 3 trial in early alzheimer’s disease. *J. Prev. Alzheimers Dis.* 10 771–777. 10.14283/jpad.2023.123 37874099

[B9] Daly C. H. Neupane B. Beyene J. Thabane L. Straus S. E. Hamid J. S. (2019). Empirical evaluation of sucra-based treatment ranks in network meta-analysis: Quantifying robustness using cohen’s kappa. *Bmj Open* 9:e24625. 10.1136/bmjopen-2018-024625 31492773 PMC6731799

[B10] Dubois B. Villain N. Frisoni G. B. Rabinovici G. D. Sabbagh M. Cappa S. (2021). Clinical diagnosis of alzheimer’s disease: Recommendations of the international working group. *Lancet Neurol.* 20 484–496. 10.1016/S1474-4422(21)00066-1 33933186 PMC8339877

[B11] Egger M. Davey S. G. Schneider M. Minder C. (1997). Bias in meta-analysis detected by a simple, graphical test. *Bmj* 315 629–634. 10.1136/bmj.315.7109.629 9310563 PMC2127453

[B12] Feng Y. Wang X. (2012). Antioxidant therapies for alzheimer’s disease. *Oxid. Med. Cell. Longev.* 2012:472932. 10.1155/2012/472932 22888398 PMC3410354

[B13] Gao Y. Qiu Y. Yang Q. Tang S. Gong J. Fan H. (2023). Repetitive transcranial magnetic stimulation combined with cognitive training for cognitive function and activities of daily living in patients with post-stroke cognitive impairment: A systematic review and meta-analysis. *Ageing Res. Rev.* 87:101919. 10.1016/j.arr.2023.101919 37004840

[B14] Gomes M. A. Akiba H. T. Gomes J. S. Trevizol A. P. de Lacerda A. Dias A. M. (2019). Transcranial direct current stimulation (tdcs) in elderly with mild cognitive impairment: A pilot study. *Dement. Neuropsychol.* 13 187–195. 10.1590/1980-57642018dn13-020007 31285793 PMC6601303

[B15] Hanslmayr S. Axmacher N. Inman C. S. (2019). Modulating human memory via entrainment of brain oscillations. *Trends Neurosci.* 42 485–499. 10.1016/j.tins.2019.04.004 31178076

[B16] Hausman H. K. Alexander G. E. Cohen R. Marsiske M. DeKosky S. T. Hishaw G. A. (2023). Primary outcome from the augmenting cognitive training in older adults study (act): A tdcs and cognitive training randomized clinical trial. *Brain Stimul.* 16 904–917. 10.1016/j.brs.2023.05.021 37245842 PMC10436327

[B17] He F. Li Y. Li C. Fan L. Liu T. Wang J. (2021). Repeated anodal high-definition transcranial direct current stimulation over the left dorsolateral prefrontal cortex in mild cognitive impairment patients increased regional homogeneity in multiple brain regions. *PLoS One* 16:e256100. 10.1371/journal.pone.0256100 34388179 PMC8363005

[B18] Huang Y. Z. Lu M. K. Antal A. Classen J. Nitsche M. Ziemann U. (2017). Plasticity induced by non-invasive transcranial brain stimulation: A position paper. *Clin. Neurophysiol.* 128 2318–2329. 10.1016/j.clinph.2017.09.007 29040922

[B19] Hutton B. Salanti G. Caldwell D. M. Chaimani A. Schmid C. H. Cameron C. (2015). The prisma extension statement for reporting of systematic reviews incorporating network meta-analyses of health care interventions: Checklist and explanations. *Ann. Intern. Med.* 162 777–784. 10.7326/M14-2385 26030634

[B20] Islam N. Hashem R. Gad M. Brown A. Levis B. Renoux C. (2023). Accuracy of the montreal cognitive assessment tool for detecting mild cognitive impairment: A systematic review and meta-analysis. *Alzheimers Dement.* 19 3235–3243. 10.1002/alz.13040 36934438

[B21] Jiang L. Cui H. Zhang C. Cao X. Gu N. Zhu Y. (2020). Repetitive transcranial magnetic stimulation for improving cognitive function in patients with mild cognitive impairment: A systematic review. *Front. Aging Neurosci.* 12:593000. 10.3389/fnagi.2020.593000 33519418 PMC7842279

[B22] Kim J. Kim H. Jeong H. Roh D. Kim D. H. (2021). Tacs as a promising therapeutic option for improving cognitive function in mild cognitive impairment: A direct comparison between tacs and tdcs. *J. Psychiatr. Res.* 141 248–256. 10.1016/j.jpsychires.2021.07.012 34256276

[B23] Kuo C. W. Chang M. Y. Liu H. H. He X. K. Chan S. Y. Huang Y. Z. (2021). Cortical electrical stimulation ameliorates traumatic brain injury-induced sensorimotor and cognitive deficits in rats. *Front. Neural Circuits* 15:693073. 10.3389/fncir.2021.693073 34194304 PMC8236591

[B24] Larson E. B. (2018). Guideline: In patients with mild cognitive impairment, the aan recommends regular exercise and no drugs or supplements. *Ann. Intern. Med.* 168:JC38. 10.7326/ACPJC-2018-168-8-038 29677245

[B25] Lissek V. Suchan B. (2021). Preventing dementia? Interventional approaches in mild cognitive impairment. *Neurosci. Biobehav. Rev.* 122 143–164. 10.1016/j.neubiorev.2020.12.022 33440197

[B26] Liu H. Wu M. Huang H. Chen X. Zeng P. Xu Y. (2024). Comparative efficacy of non-invasive brain stimulation on cognition function in patients with mild cognitive impairment: A systematic review and network meta-analysis. *Ageing Res. Rev.* 101:102508. 10.1016/j.arr.2024.102508 39303877

[B27] Ni Y. Xiao Y. Shen B. Sun Y. M. Zhao J. Wu B. (2025). Impact of deep brain stimulation on cognitive impairment in parkinson’s disease: A retrospective longitudinal study. *Neurotherapeutics* 22:e561. 10.1016/j.neurot.2025.e00561 40000338 PMC12047459

[B28] Nissim N. R. McAfee D. C. Edwards S. Prato A. Lin J. X. Lu Z. (2023). Efficacy of transcranial alternating current stimulation in the enhancement of working memory performance in healthy adults: A systematic meta-analysis. *Neuromodulation* 26 728–737. 10.1016/j.neurom.2022.12.014 36759231 PMC10257732

[B29] Pawelec P. Ziemka-Nalecz M. Sypecka J. Zalewska T. (2020). The impact of the cx3cl1/cx3cr1 axis in neurological disorders. *Cells* 9:2277. 10.3390/cells9102277 33065974 PMC7600611

[B30] Petersen R. C. Lopez O. Armstrong M. J. Getchius T. Ganguli M. Gloss D. (2018). Author response: Practice guideline update summary: Mild cognitive impairment: Report of the guideline development, dissemination, and implementation subcommittee of the american academy of neurology. *Neurology* 91 373–374. 10.1212/WNL.0000000000006042 30126885

[B31] Puhan M. A. Schunemann H. J. Murad M. H. Li T. Brignardello-Petersen R. Singh J. A. (2014). A grade working group approach for rating the quality of treatment effect estimates from network meta-analysis. *Bmj* 349:g5630. 10.1136/bmj.g5630 25252733

[B32] Reinhart R. Nguyen J. A. (2019). Working memory revived in older adults by synchronizing rhythmic brain circuits. *Nat. Neurosci.* 22 820–827. 10.1038/s41593-019-0371-x 30962628 PMC6486414

[B33] Rouse B. Chaimani A. Li T. (2017). Network meta-analysis: An introduction for clinicians. *Intern. Emerg. Med.* 12 103–111. 10.1007/s11739-016-1583-7 27913917 PMC5247317

[B34] Salech F. SanMartin C. D. Concha-Cerda J. Romero-Hernandez E. Ponce D. P. Liabeuf G. (2022). Senescence markers in peripheral blood mononuclear cells in amnestic mild cognitive impairment and Alzheimer’s disease. *Int. J. Mol. Sci.* 23:9387. 10.3390/ijms23169387 36012652 PMC9409141

[B35] Sanford A. M. (2017). Mild cognitive impairment. *Clin. Geriatr. Med.* 33 325–337. 10.1016/j.cger.2017.02.005 28689566

[B36] Shamseer L. Moher D. Clarke M. Ghersi D. Liberati A. Petticrew M. (2015). Preferred reporting items for systematic review and meta-analysis protocols (prisma-p) 2015: Elaboration and explanation. *Bmj* 350:g7647. 10.1136/bmj.g7647 25555855

[B37] Sterne J. Savovic J. Page M. J. Elbers R. G. Blencowe N. S. Boutron I. (2019). Rob 2: A revised tool for assessing risk of bias in randomised trials. *Bmj* 366:l4898. 10.1136/bmj.l4898 31462531

[B38] Tang Y. Xing Y. Sun L. Wang Z. Wang C. Yang K. (2024). Transcranial alternating current stimulation for patients with mild Alzheimer’s disease (transform-ad): A randomized controlled clinical trial. *Alzheimers Res. Ther.* 16:203. 10.1186/s13195-024-01570-0 39267112 PMC11395938

[B39] Traikapi A. Konstantinou N. (2021). Gamma oscillations in Alzheimer’s disease and their potential therapeutic role. *Front. Syst. Neurosci.* 15:782399. 10.3389/fnsys.2021.782399 34966263 PMC8710538

[B40] Tseng P. T. Chen Y. W. Zeng B. Y. Zeng B. S. Hung C. M. Sun C. K. (2023). The beneficial effect on cognition of noninvasive brain stimulation intervention in patients with dementia: A network meta-analysis of randomized controlled trials. *Alzheimers Res. Ther.* 15:20. 10.1186/s13195-023-01164-2 36698219 PMC9875424

[B41] van Olst L. Simonton B. Edwards A. J. Forsyth A. V. Boles J. Jamshidi P. (2025). Microglial mechanisms drive amyloid-beta clearance in immunized patients with Alzheimer’s disease. *Nat. Med.* 31 1604–1616. 10.1038/s41591-025-03574-1 40050704 PMC12092304

[B42] Vints W. Levin O. van Griensven M. Vlaeyen J. Masiulis N. Verbunt J. (2024). Neuromuscular electrical stimulation to combat cognitive aging in people with spinal cord injury: Protocol for a single case experimental design study. *BMC Neurol.* 24:197. 10.1186/s12883-024-03699-9 38862912 PMC11165793

[B43] Wang L. F. Liang W. D. Wang B. Y. Guo M. L. Zhou J. S. Chen L. (2022). Transcutaneous electrical acupoint stimulation for reducing cognitive dysfunction in lumbar spine surgery: A randomized, controlled trail. *Front. Aging Neurosci.* 14:1034998. 10.3389/fnagi.2022.1034998 36545028 PMC9760873

[B44] Wang L. Zhang J. Guo C. He J. Zhang S. Wang Y. (2022). The efficacy and safety of transcutaneous auricular vagus nerve stimulation in patients with mild cognitive impairment: A double blinded randomized clinical trial. *Brain Stimul.* 15 1405–1414. 10.1016/j.brs.2022.09.003 36150665

[B45] Wang T. Yan S. Lu J. (2023). The effects of noninvasive brain stimulation on cognitive function in patients with mild cognitive impairment and alzheimer’s disease using resting-state functional magnetic resonance imaging: A systematic review and meta-analysis. *CNS Neurosci. Ther.* 29 3160–3172. 10.1111/cns.14314 37349974 PMC10580344

[B46] Wang W. Chen C. Wang Q. Ma J. G. Li Y. S. Guan Z. (2024). Electroacupuncture pretreatment preserves telomerase reverse transcriptase function and alleviates postoperative cognitive dysfunction by suppressing oxidative stress and neuroinflammation in aged mice. *CNS Neurosci. Ther.* 30:e14373. 10.1111/cns.14373 37501354 PMC10848091

[B47] Ward A. Arrighi H. M. Michels S. Cedarbaum J. M. (2012). Mild cognitive impairment: Disparity of incidence and prevalence estimates. *Alzheimers Dement.* 8 14–21. 10.1016/j.jalz.2011.01.002 22265588

[B48] Wu J. Ishikawa M. Zhang J. Hashimoto K. (2010). Brain imaging of nicotinic receptors in Alzheimer’s disease. *Int. J. Alzheimers Dis.* 2010:548913. 10.4061/2010/548913 21253523 PMC3022172

[B49] Yang A. Pechlivanoglou P. Aoyama K. (2022). Interpreting and assessing confidence in network meta-analysis results: An introduction for clinicians. *J. Anesth.* 36 524–531. 10.1007/s00540-022-03072-5 35641661 PMC9338903

[B50] Zhou Y. Hu C. Mao C. Li S. Cui Y. Qian Y. (2022). Electroacupuncture ameliorates tibial fracture-induced cognitive dysfunction by elevating alpha7nachr expression and suppressing mast cell degranulation in the hippocampus of rats. *Evid. Based Complement Alternat. Med.* 2022:3182220. 10.1155/2022/3182220 35463074 PMC9019405

[B51] Zoefel B. Ten O. S. Sack A. T. (2018). The involvement of endogenous neural oscillations in the processing of rhythmic input: More than a regular repetition of evoked neural responses. *Front. Neurosci.* 12:95. 10.3389/fnins.2018.00095 29563860 PMC5845906

