# Supplementary material for: Cognitive improvement by non-pharmacological electrical stimulation modalities in mild cognitive impairment: a protocol for systematic review and network meta-analysis
Source: Front Aging Neurosci. 2026 Mar 11;18:1752516. doi: 10.3389/fnagi.2026.1752516 (PMC13013000; doi:10.3389/fnagi.2026.1752516)
Supplement: Supplementary file 2 [file Table_2.docx]

| Order | Strategy |
| --- | --- |
| #1 | Search: “Mild cognitive impairment”[Mesh] |
| #2 | Search: (Cognitive impairment[Title/Abstract]) OR (Cognitive decline[Title/Abstract]) OR (cognitive impairment[Title/Abstract]) OR (Cognitive dysfunction[Title/Abstract]) OR (MCI [Title/Abstract] ) OR (MCI[Title/Abstract])) |
| #3 | #1 OR #2 |
| #4 | Search: “Electrical stimulation”[Mesh] |
| #5 | Search: (Electrical Stimulation[Title/Abstract]) OR (Electrical Stimulations[Title/Abstract]) OR (Stimulation, Electrical[Title/Abstract]) OR (Stimulations, Electrical[Title/Abstract]) OR (Stimulation, Electric[Title/Abstract]) OR (Electric Stimulations[Title/Abstract]) OR (Stimulations, Electric[Title/Abstract]) OR (transcranial direct current stimulation[Title/Abstract]) OR (transcranial alternating current stimulation[Title/Abstract]) OR (transcutaneous electrical acupoint stimulation [Title/Abstract]) OR (repetitive transcranial magnetic stimulation[Title/Abstract]) OR (electroacupuncture [Title/Abstract]) OR (cortical electrical stimulation[Title/Abstract]) OR (deep brain stimulation[Title/Abstract]) OR (neuromuscular electrical stimulation[Title/Abstract]) OR (transcutaneous auricular vagus nerve stimulation[Title/Abstract]) |
| #6 | #4 OR #5 |
| #7 | Search: (randomized controlled trial) OR (randomized) OR (placebo) |
| #8 | #3 AND #6 AND #7 |

Supplementary Material

# S2 The search strategy for PubMed

**The search strategy for China National Knowledge Infrastructure (CNKI)**

| Strategy |
| --- |
| SU%='电刺激'+'经颅直流电刺激'+'经颅交流电刺激'+'经皮电刺激'+'重复经颅磁刺激'+'电针'+'皮质电刺激'+'深部脑刺激'+'神经肌肉电刺激'+'经皮耳迷走神经刺激' AND SU%='轻度认知障碍'+'轻度认知功能障碍'+'认知障碍'+'遗忘型轻度认知障碍'+'MCI'+'aMCI' AND TKA%='随机对照实验'+'随机对照试验'+'随机对照研究'+'随机对照'+'RCT'+'随机' |
